# Befriending the body through clothes: the role of clothing in secular and religious women’s body appreciation

**DOI:** 10.3389/fpsyg.2024.1297663

**Published:** 2024-05-30

**Authors:** Tali Stolovy

**Affiliations:** Ono Academic College, Academic College of Society and the Arts (ASA), Netanya, Israel

**Keywords:** body appreciation, clothing functions, body image, feministic, religiosity

## Abstract

**Background:**

Women invest in their appearance through clothes, and the way they view their bodies translates into how they choose to dress. Nonetheless, body image research often overlooks the role of clothing in fostering body appreciation. This study examined the impact of a psychoeducational feministic course on the sociology and psychology of attire, on students’ clothing functions and body appreciation.

**Methods:**

The participants were 114 female MA students (47 secular, 67 religious) between the ages of 24 and 64 who completed the Body Appreciation Scale and Function of Clothing scale at the beginning and end of the course.

**Results:**

The results support the contribution of the course to changes in participants’ clothing functions and improvement in their body appreciation *F*(1,96) = 32.33, *p* < 0.001, partial Eta squared = 0.25. Surprisingly, religiousness had no impact on the results.

**Discussion:**

This research contributes to the field of positive body image by presenting the potential role of clothing in fostering body appreciation among women. It demonstrates the benefits of investing in clothing that are less driven by external standards and more by the expression of valued aspects of the self.

## 1 Introduction

Women in many cultures, particularly in Western cultures, are socialized to prioritize beauty and fashion (Rudd and Lennon, 2000). The way women perceive and interpret appearance-related messages significantly impacts their body image, i.e., their positive and negative perceptions and attitudes toward their body and appearance (Cash, 2004). Body image concerns are prevalent globally, including in the Middle East, due to strong pressures to conform to appearance ideals (Rodgers et al., 2023). Given the detrimental effects on global mental and physical health, body dissatisfaction becomes a focal point for numerous interventions and prevention programs. Evidence suggests that cultivating positive body image should also be a key intervention target (Guest et al., 2019). Positive body image entails respecting, honoring, loving, and expressing gratitude toward one’s body. Most research in the field of positive body image focuses on body appreciation (Tylka and Piran, 2019).

Body image research shows that many aspects of how women view their bodies translate into their choices of attire (Tiggemann and Lacey 2009). Shim et al. (1990) found that women dissatisfied with their bodies harbored negative attitudes toward clothing, exhibited less confidence in their clothing selection, and were less inclined to be fashion innovators. Frith and Gleeson (2008) noted that women often use clothing to alter or manage their body’s appearance and their feelings of bodily anxiety, particularly on what they define as “fat days.” Similarly, Kwon and Parham (1994) found that women tended to select clothes more for camouflage purposes and less for individual self-expression when they felt “fat” as opposed to when they felt thinner. In their study, a higher body mass index (BMI) was related to camouflage. Tiggemann and Andrew (2012) also found that body weight is positively correlated with seeking clothing for camouflage. Further, their findings showed that the importance individuals placed on their physical appearance (i.e., self-objectification) correlated positively with choosing clothes for fashion, but negatively with seeking clothing for comfort. To conclude, research demonstrates that body image can influence women’s clothing practices (Kaiser, 1985; Tiggemann, 2004; Tiggemann and Andrew, 2012).

The impact of clothing on body image is multifaceted. Clothing may serve as an “appearance-fixing” behavior, associated with greater body dissatisfaction, as individuals seek to conform to beauty ideals (Cash et al., 2005). In such cases, clothing becomes an externally driven pursuit, reflecting the desire to align with societal beauty standards. Clothing also functions as a tool for impression management and may indicate self-objectification (Tiggemann and Andrew, 2012). In other cases, clothing may serve as a coping strategy that allows a sense of mastery over the body and a shift to a more positive body image state (Frith and Gleeson, 2008). Qualitative research found that women use clothing to befriend the body, to love it as it is with its imperfections (Masuch and Hefferon, 2014). Cash (2008) coined the term “flexible groomer” to describe individuals who enjoy using clothing for mastery and pleasure rather than rigidly adhering to socially acceptable appearance norms.

There is evidence that clothing practices can directly shape cognitive processes. Experimental research has demonstrated this concept of ‘enclothed cognition’; when donning a white lab coat, participants performance improved significantly on attention- related tasks (Adam and Galinsky, 2012). Clothing may contribute to perceived life quality (Sontag and Lee, 2004) by expressing one’s identity (Rocamora, 2017; Valaei and Nikhashemi, 2017; McNeill and Venter, 2019), providing comfort and safety (Kwon and Shim, 1999; Rahman et al., 2021), and altering mood states (Moody et al., 2010; Koksal, 2014; McNeill and Venter, 2019).

The therapeutic potential of clothing was identified by a select group of researchers who use the term ‘fashion therapy’ to describe the positive psychological effects of clothing on the wearer. They focus on actual changes in appearance as noted by fashion experts (Callis, 1982; Lee et al., 2020). This approach originated in psychiatric wards for patients with poor hygiene and grooming skills (Roach and Eicher, 1965; Wong et al., 1988). However, therapeutic utilization of fashion requires an understanding of the complex relationship between women and clothing (Guy and Banim, 2000), as well as societal pressures on women’s appearance (Tiggemann, 2004). If clothing practices prioritize attaining fashionable standards, it may exacerbate self-objectification and body dissatisfaction (Lemma, 2014; Engeln and Zola, 2021). The positive aspect of clothing lies in the flexibility and playfulness that Cash (2008) defined as “flexible grooming.” From a feminist perspective, it is the difference between dressing for the male gaze and dressing out of a personal narrative (Braizaz, 2018).

Body image interventions generally aim to prevent body image disturbances while promoting body appreciation. These interventions, often implemented in educational settings, include components such as media literacy, body acceptance, a broad conceptualization of beauty, emotional regulation skills, self-compassion, and mindfulness training (Guest et al., 2019; Yager, 2019). Additionally, body image interventions are informed by feminist perspectives, seeking to resist objectification through critiques of gender roles and stereotypes (Murnen and Seabrook, 2012). Feminist ideologies were found to have a positive effect on body image (Snyder and Hasbrouck, 1996; Peterson et al., 2006). The literature review for this research failed to uncover any findings regarding the use of clothing as a modality within existing body image interventions (e.g., Guest et al., 2019).

Israel presents an opportunity to study the relationship between clothing and body image in a diverse society with varying religious identities. The Jewish population in Israel can be categorized into three religious sub-groups: Orthodox and ultra-Orthodox, Modern Orthodox, and secular. These groups differ in terms of clothing practices and views on female modesty (Geller et al., 2020). Modesty norms entail gender separation from a young age and strict standards of dress and behavior. Orthodox and Ultra-Orthodox women are instructed to cover most of their bodies, including elbows, knees, collarbones, and toes. Clothing must be unobtrusive, resulting in common colors such as white, black, gray, dark blue, and brown in the Orthodox female wardrobe. The Modern orthodox dress code is less strict and meticulous (Taragin-Zeller, 2014). Moreover, the Modern Orthodox society is more exposed to western media in an attempt to synthesize compliance with Jewish law with the secular modern world (Seigelshifer and Hartman, 2011).

Within the cultural context of body image, various aspects of religious practices and affiliation have been identified as protective factors against body image dissatisfaction (Mussap, 2009; Handelzalts et al., 2017). Modest dress codes have been associated with lower experiences of objectification, allowing women to affirm themselves as human beings rather than solely as sexual objects. Consequently, this may serve as a buffering mechanism against societal appearance pressures (Mussap, 2009). Indeed, Handelzalts et al. (2017) found that ultra-Orthodox Jewish women maintained more positive attitudes toward their bodies. Media exposure has been suggested as a mediator for the relationship between religiosity levels and body image (Geller et al., 2020), given its established detrimental role in promoting body- and weight-dissatisfaction (Halliwell, 2013). However, the relationship between religion and positive body image needs to be further examined, as contrary findings suggest no significant correlation between religion and body image concerns (Akrawi et al., 2015).

The present study aims to investigate the potential contribution of clothing to body appreciation among religious and secular adult women in Israel. Specifically, it focuses on five clothing functions: camouflage, comfort, assurance, fashion, and individuality. These functions are explored within a context similar to existing body image interventions, particularly those informed by feminist perspective.

### 1.1 The present study

This study explored the relationship between clothing, body appreciation, and religiosity. The study included religious and secular adult women who enrolled in an elective course named “Styling as Self-Expression” as part of their MA in Society and the Arts. The course has been part of the MA program for 4 years before the research was conducted and was not specially designed for the study. The course outline, contents and aims are presented in Table 1. This psychoeducational course discusses the sociology and psychology of attire from a feminist perspective, placing emphasis on cultivating body appreciation. The course aimed to present a novel view of clothing as a societal construct with significant psychological implications that could benefit body image and overall well-being. It comprised 13 lessons, each lasting 2 academic hours, and encouraged participants to explore their self-expression and playfulness with clothes. While homework was voluntary and unsupervised, participation was mandatory.

**Table 1:** “Styling as self-expression” – course outline, contents^*^, and aims.

Lesson	Content	Psychoeducational aim
1	Introduction to concepts and theories on the psychology of clothes	Restructuring the meaning of fashion for women and exploring emotional meaning of clothing
2–3	Clothing functions (Kwon and Parham, 1994)	Guided self-discovery of personal and social motives for daily choices of clothing (personal and social identities are interdependent)
4–5	Objectification theory (Fredrickson and Roberts, 1997)	Feministic perspective on beauty standards, gender expectations and risks of self-objectification
6–7	Body image (Cash, 2008)	Fostering body acceptance through “flexible grooming”; Adjusting clothes to personal needs and preferences and not vice versa; Critical thinking about clothing size and fashion’s exclusivity; Focusing on the body’s assets and not imperfections; Media literacy around fashion, style and beauty expectations
8	Self compassion (Neff, 2003; Gilbert, 2015)	Befriending the body and self; Minimizing self-criticism in the context of the gaze in the mirror; Fostering self-compassion skills for moments of fluctuations in body image
9	Clothing styles and personality traits (Stolovy, 2021)	Clothing as a path for self-expression rather than the exclusive focus on body presentation – what kind of story do we want to express through clothes?
10	Enclothed cognition (Adam and Galinsky, 2012)	Utilizing the psychological effect of clothes from a point of agency and empowerment; Expressing strength of character through symbolic meaning of clothes
11	Openness to experience (Costa and McCrae, 1992)	Fostering playfulness with clothes; Guided self discovery of different expressions through clothes; The courage to challenge societal and personal expectations around styling (dressing in different ways)
12	Emotional regulation through clothing practices	Personal exploration of emotional effects of colors, fabrics, shapes, and tactical qualities of attire; Striving for positive body connection and comfort through clothing choices; Enhancing positive emotions and wellbeing through colors
13	Psychology of clothing consumption (Benson, 2000)	Exploring motives of shopping; Fostering mindfulness in shopping; Understanding the internal dialog while measuring clothes in stores

^*^References presented in this table are basis for the course content.

Given the diverse religiosity within the student population, including ultra-Orthodox and secular students, the study aimed to examine the effect of the course on body appreciation while considering the participants’ level of religiosity.

It was hypothesized that: (a) The course would increase participants’ levels of body appreciation; (b) Religious students would demonstrate higher levels of body appreciation at both time points; (c) The course would contribute to changes in the clothing functions of participants, resulting in lower levels of camouflage and higher levels of individuality and assurance.

## 2 Methods

### 2.1 Participants and procedure

To ensure the ethical treatment of human participants, this study was carried out according to the ethics principles of the Academic College of Society and the Arts and its institutional research committee. Participation was entirely voluntary, and consent was obtained in advance. The participants were 114 female MA students (47 secular, 67 religious) aged between 24 and 64 (*M* = 42.7, *SD* = 9.5). The participants were invited to fill out self-report questionnaires confidentially at two time points (at the end of the first lesson and immediately after the last one). They completed the questionnaires anonymously and provided a 4-digit number so that their questionnaires from the two time points could be aligned. The participants took part voluntarily and were not remunerated for their time. There were no exclusion criteria, but incomplete forms or those filled out at only one time point were excluded from the research. Additionally, 27 students did not volunteer to participate and were thus excluded from the sample. Missing data accounted for 2–13% of the dataset. Two variables, BMI and comfort, had substantial missing data. Little’s MCAR test: Chi-Square = 193.123, DF = 68, Sig. = 0.000 with the exclusion of these two variables, Little’s test was not significant, indicating that for BMI and comfort, the missing data was not random. This issue is further addressed in the discussion.

### 2.2 Measures

#### 2.2.1 Function of clothing scale

Functions of clothing were assessed using items developed by Kwon and Parham (1994). This scale measures the choice of clothing for its comfort, camouflage, assurance, fashion, and individuality functions. The items are assessed on a Likert scale ranging from 1 (not at all agree) to 5 (very much agree) in response to the stem “I tend to select…” The five categories are: Fashion (e.g., “clothes that are stylish”; 3 items), Camouflage (e.g., “clothes that camouflage my figure problems”; 3 items), Assurance (e.g., “clothes that give me self-confidence”; 5 items), Individuality (e.g., “clothes that make me distinctive”; 3 items), and Comfort (e.g., “clothes that are comfortable”; 1 item). The scale was translated to Hebrew using a translation/back translation procedure by the author and a native English speaker and used in former study (Stolovy, 2021). In the present sample, Cronbach’s alpha was 0.72 at time 1 and 0.71 at time 2.

#### 2.2.2 Body appreciation

Body appreciation was assessed by the Body Appreciation Scale (BAS) developed by Avalos et al. (2005). This 13-item scale contains items addressing the appreciation, acceptance, respect, and attention given to one’s body (e.g., “I respect my body,” “Despite my flaws, I accept my body for what it is”). Responses are on a 5-point Likert scale (1 = never, 5 = always) and averaged, with higher scores reflecting greater body appreciation. The scale was translated to Hebrew using a translation/back translation procedure by the author and a native English speaker. Tylka and Wood-Barcalow (2015) report Cronbach’s alpha as being above 0.90, indicating high internal consistency. In the present sample, Cronbach’s alpha was 0.87 at time 1 and 0.91 at time 2.

#### 2.2.3 Sociodemographic data

Each participant was asked to indicate her age, height and weight for calculation of body mass index (BMI), place of residence, marital status, health, financial status and religious denomination.

### 2.3 Statistical analyses

A power analysis was conducted to determine the required sample size for detecting the hypothesized effect. Based on an expected effect size of *d* = 0.30, an alpha level of 0.05, and a desired power of 0.80, a minimum sample size of 90 participants was calculated using G*Power software (Faul et al., 2007). After excluding missing data, the final sample size was 114 participants. The data were analyzed with SPSS 19.0 software. The relationships between clothing functions, body appreciation, and background data (i.e., age, marital status, health and financial statuses, and places of residence) were analyzed using Pearson’s correlations and chi-square tests. A two way MANOVA (multivariate analysis of variance) was used to explore the combined effect of participation in the course (effect of time) and religious affiliation (inter-group differences) on the research variables, i.e., clothing functions and body appreciation.

## 3 Results

### 3.1 Characteristics of the sample

The sample comprised 114 women from urban and rural areas in Israel; the participants’ ages ranged from 24 to 64 (*M* = 42.7, *SD* = 9.5). Descriptive data is presented in Table 2. As can be seen, approximately half of the sample consider themselves to be religious (*N* = 67, 58.8%) and the rest are secular (*N* = 47, 41.2%). Most of the sample are married (*N* = 89, 78.1%) with children (*N* = 99, 86.8%). BMI scores ranged from 16.18 to 54.43 (*M* = 24.9, *SD* = 5.06). Chi-squared tests found no association between descriptive data, clothing functions and body appreciation.

**Table 2:** Descriptive statistics for sample characteristics (*N* = 114).

Variable	Mean, *SD*
Age	42.7, 9.5
Height (cm)	162.8, 5.4
Weight (kg)	66.1, 13.7
BMI	24.88, 5.06
Variable	N^*^, % of the sample
Marital status	
Single	15, 13.2%
Married or living as married	89, 78.1%
Divorced	8, 7%
Widowed	1, 0.9%
Children	
Yes	99, 86.8%
No	15, 13.2%
Religious affiliation	
Secular	47, 41.2%
Religious	67, 58.8%
Financial status	
Very good	24, 21.1%
Good	48, 42.1%
Moderate	41, 36.0%
Not good	1, 0.9%
Health condition	
Very good	51, 44.7%
Good	53, 46.5%
Moderate	10, 8.8%
Place of residence	
North country	38, 33.3%
Central country	74, 65%
South country	1, 0.9%

^*^N presented excluding missing data.

### 3.2 Body appreciation and clothing functions

The patterns of correlations between body appreciation (BAS), clothing functions, and BMI are shown in Tables 3, 4. As can be seen, at time 1, BAS is positively correlated with assurance and negatively correlated with BMI and camouflage. It is interesting that, at time 2, BAS is positively correlated with all clothing functions including camouflage. BMI presents the expected correlations with clothing functions; at time 1, it is positively correlated with camouflage, and negatively correlated with assurance, fashion, and BAS. At time 2, BMI is no longer correlated with comfort or fashion. At time 1, fashion is negatively correlated with BMI, but at time 2, it is not. However, it correlates with all clothing functions. All correlations are presented in Tables 3, 4 and will be thoroughly discussed.

**Table 3:** Descriptive statistics and correlations of body appreciation (BAS) and clothing functions – time 1 (*N* = 114).

Variables	*M*	*SD*	1	2	3	4	5	6	7
1. BAS	3.55	0.63	–	−0.52^***^	0.15	0.12	0.28^**^	−0.44^***^	−0.04
2. BMI	24.88	5.06		–	−0.20^*^	−0.09	−0.22^*^	0.34^***^	0.25^*^
3. Fashion	2.85	0.99			–	0.52^***^	0.58^***^	−0.06	−0.18^*^
4. Individuality	3.0	0.93				–	0.54^***^	0.02	−0.10
5. Assurance	3.58	0.67					–	−0.13	0.15
6. Camouflage	3.11	1.09						–	0.28^**^
7. Comfort	4.15	0.85							–

^*^Indicates significance at *p < 0.05, ^**^p < 0.01, ^***^p < 0.001.

**Table 4:** Descriptive statistics and correlations of body appreciation (BAS) and clothing functions – time 2 (*N* = 114).

Variables	*M*	*SD*	1	2	3	4	5	6	7
1. BAS	4.38	1.6	–	−0.47^***^	0.86^***^	0.86^***^	0.92^***^	0.80^***^	0.82^***^
2. BMI	24.84	5.02		–	−0.47	−0.19	−0.26^*^	0.52^***^	0.05
3. Fashion	3.59	1.99			–	0.88^***^	0.93^***^	0.79^***^	0.75^***^
4. Individuality	3.82	1.91				–	0.91^***^	0.79^***^	0.72^***^
5. Assurance	4.38	1.63					–	0.82^***^	0.81^***^
6. Camouflage	3.49	1.99						–	0.82^***^
7. Comfort	4.62	1.67							–

^*^Indicates significance at *p < 0.05, ^**^p < 0.01, ^***^p < 0.001.

### 3.3 Effect of course participation on clothing functions and body appreciation

A multivariate analysis of variance (MANOVA) procedure was employed to investigate potential differences in BAS and clothing functions across the two measurement times, while considering participants’ religious affiliation. Table 5 presents estimated marginal means and MANOVA results for the main univariate effects of time across outcomes.

**Table 5:** Estimated marginal means and main effects of time for the research variables (*N* = 114).

	EMM Time 1	EMM Time 2	Main effect of time	*p-*value	*η^2^_p_*
BAS	3.56 (0.07)	3.86 (0.06)	*F*(1,96) = 32.32	<0.001	0.25
BMI	24.90 (0.53)	24.90 (0.52)	*F*(1,96) = 0.00	0.96	0.00
Comfort	4.24 (0.08)	4.19 (0.09)	*F*(1,96) = 0.24	0.62	0.00
Camouflage	3.12 (0.12)	2.90 (0.08)	*F*(1,96) = 4.36	<0.05	0.04
Assurance	3.62 (0.07)	3.90 (0.06)	*F*(1,96) = 30.55	<0.001	0.24
Fashion	2.80 (0.09)	3.01 (0.09)	*F*(1,96) = 8.95	<0.01	0.09
Individuality	2.99 (0.09)	3.24 (0.09)	*F*(1,96) = 10.07	<0.01	0.10

EMM, estimated marginal means; BAS, body appreciation scale; BMI, body mass index.

The multivariate effect of time was significant [*F*(7,90) = 7.44, *p* < 0.001, partial Eta squared = 0.37]. BAS scores showed an increase between the two measurement times [*F*(1,96) = 32.33, *p* < 0.001, partial Eta squared = 0.25], while BMI remained unchanged [*F*(1,96) = 0.003, *p* = 0.96]. However, average values of camouflage decreased [*F*(1,96) = 4.36, *p* = 0.04, partial Eta squared = 0.04], while assurance [*F*(1,96) = 30.55, *p* < 0.001, partial Eta squared = 0.24], fashion [*F*(1,96) = *p* = 0.004, partial Eta squared = 0.08], and individuality [*F*(1,96) = 10.08, *p* = 0.002, Eta squared = 0.10] increased between the two time points.

The MANOVA indicated that the effect of religiosity was not significant: *F*(7,90) = 1.70, *p* = 0.12. Table 6 provides means and standard deviations separately for secular women and religious women.

**Table 6:** Mean and standard deviations of research variables according to religion (*N* = 114).

	Secular (*N* = 47)		Religious (*N* = 67)	
	Time 1 *M* (*SD*)	Time 2 *M* (*SD*)	Time 1 *M* (*SD*)	Time 2 *M* (*SD*)
BAS	3.55 (0.67)	3.87 (0.54)	3.56 (0.61)	3.86 (0.64)
BMI	25.19 (5.81)	25.03 (5.36)	24.61 (4.69)	24.76 (4.80)
Comfort	4.44 (0.67)	4.37 (0.70)	4.04 (0.89)	4.02 (0.96)
Camouflage	3.09 (0.93)	2.88 (0.92)	3.15 (1.30)	2.93 (0.73)
Assurance	3.72 (0.62)	3.97 (0.61)	3.52 (0.66)	3.85 (0.61)
Fashion	2.76 (0.98)	3.10 (0.98)	2.82 (0.93)	2.92 (0.86)
Individuality	2.97 (0.92)	3.11 (0.88)	3.01 (0.96)	3.37 (0.88)

BAS, body appreciation scale; BMI, body mass index.

## 4 Discussion

This study investigated the influence of clothing on body appreciation among both secular and religious women. Specifically, it explored how a psychoeducational feminist course focusing on the sociology and psychology of attire affected students’ clothing functions and body appreciation. Overall, the findings suggest that the course may have had beneficial impact in altering participants’ clothing functions and enhancing their body appreciation. Surprisingly, participants’ religious domination did not appear to effect the outcomes.

Consistent with prior research (Shim et al., 1990; Kwon and Parham, 1994; Tiggemann and Lacey, 2009), the study revealed a correlation between body dissatisfaction and the tendency to use clothes for camouflage. At the initial time point (time 1) of the study, participants demonstrated a higher inclination toward camouflage as their BMI increased. This inclination to conceal the body was associated with lower body appreciation scores. Additionally, Fashion was inversely related to BMI, indicating that participants perceived fashionable clothing as associated with a thinner body. This highlights the intricate relationship women have with fashion, where they often equate it with a slim figure and strive to conform to societal standards (Apeagyei, 2008). Notably, the fashion industry tends to offer limited choices and creates a discouraging shopping environment for individuals who do not fit into standardized, small apparel sizes (Colls, 2004; Christel, 2014; Peters, 2014). The findings indicate that participants gained more confidence with fashion and clothing over time, regardless of their body size. Fashion became correlated with all clothing functions and no longer correlated with body size. This enhanced confidence aligns with one of the primary goals of the course, which is fostered by its feminist perspective. Feminist interventions take a proactive stance against societal pressures related to beauty standards (Murnen and Seabrook, 2012) and employ psychoeducation to identify and counter harmful messages regarding appearance and gender roles (Tylka and Piran, 2019).

The primary message of the course emphasized that clothing should be tailored to suit the individual wearer, rather than expecting the wearer to conform to clothing standards. The course included psychoeducation to address common challenges women face in their interactions with fashion. For many women, the act of shopping for and trying on clothes can serve as a stark reminder that their bodies do not align with societal norms (Tiggemann and Lacey, 2009). Therefore, the course aimed to transform the fashion experience from one of self-evaluation to one of compassion. Through tools such as media literacy, self-compassion exercises, and playful exploration of clothing, participants were encouraged to develop a sense of self-confidence in their fashion choices. The course depicted fashion and style as opportunities for individuals to express valued aspects of their identity, rather than merely showcasing their bodies.

The MANOVA results provide further support for the course’s impact on participants’ body appreciation. As body appreciation increased over time, so did participants’ levels of assurance, fashion, and individuality with clothing. It is plausible that participants felt empowered to express their individuality through fashion (Belk, 1997), and this self-expression had a positive effect on their body appreciation, consistent with findings from previous studies (Masuch and Hefferon, 2014; Stolovy, 2021).

Participants’ attitudes toward camouflage underwent an intriguing shift. Initially, at time 1, camouflage was correlated with higher BMI and comfort. However, at time 2, camouflage demonstrated multiple correlations with clothing functions. This suggests that participants may have perceived camouflage as a means of exerting control and enhancing confidence through their clothing, rather than solely as a method of concealing body flaws. This aligns with Frith and Gleeson’s (2008) research, which indicates that women utilize clothing to both conceal and highlight aspects of their bodies. For some women, the act of using clothes to conceal certain body features can lead to increased feelings of confidence. This differs from a generalized desire to conceal the body due to its size and associated feelings of dissatisfaction, which often result in negative attitudes toward clothing (e.g., Tiggemann, 2004). These findings suggest that the course encouraged participants to expand their perceptions of clothing functions, irrespective of their body size. It is important to note that weight and height reports presented substantial missing data, indicating that self-reported BMI may be biased. Women face a widespread and socially acceptable stigma associated with heavier body weight and the utility of BMI measurement may be poor.

Tiggemann and Lacey (2009) suggested that clothing choices may influence how women feel about their bodies. The present study demonstrates that, by enhancing enjoyment and flexibility with clothing, women can enhance body appreciation. As previously suggested by Frith and Gleeson (2008), clothing practices can serve not only to present body image but also to manage fluctuations in body image.

The course described above used the known positive impact of feminist ideologies on cognitive restructuring, re-labeling women’s thoughts and experiences (Srebnik and Saltzberg, 1994), and its positive effect on body image (Snyder and Hasbrouck, 1996; Peterson et al., 2006). It is different from “fashion therapy” (Callis, 1982; Lee et al., 2020; Kang and Kim, 2021) since it involves no directive guidelines for fashionable or recommended clothing. Participants were encouraged to explore different expressions through fashion and to challenge their perceptions of clothing functions. Moreover, the relationship with clothes was put within the context of attuned self-care and the bodily sensation of fabrics and textures to enhance the connection to one’s embodied self (Piran, 2016).

Finally, the similarities between secular and religious participants must be considered. Religion has been described as a protective factor for body image (Kim, 2006; Mussap, 2009), and Handelzalts et al. (2017) found that ultra-Orthodox Jewish women maintained more positive attitudes toward their bodies. It was surprising that religious participants had no advantage over their secular colleagues in terms of body appreciation and clothing functions. This similarity may be understood in light of Geller et al.’s (2020) findings among the religious community in Israel. Geller et al. (2020) found differences in positive body image between Ultra-Orthodox women compared to Modern Orthodox and secular women, but no difference between secular and Modern Orthodox women. Media exposure mediated the relationship between religiosity and body image. Modern Orthodox women are found to be more open to modern life and more exposed to Western media (Geller et al., 2020). Participants in the current study may fit the definition of Modern Orthodox: adhering to religious laws and most traditions, with an openness to modern life and Western media (Seigelshifer and Hartman, 2011). The religious participants in this study attended the course at a secular institution for higher education, alongside secular women. Their media exposure possibly resembles that of their secular colleagues, as does their body appreciation. Moreover, the relationship between religion and positive body image needs further examination, as there are findings that do not support the correlation between religion and body image concerns (Akrawi et al., 2015).

## 5 Conclusion

This study highlights the relationship between clothing and body appreciation among women in a diverse society with varying religious identities. Given the detrimental effects of body image concerns on global mental and physical health, programs and interventions that can protect or improve body image, are sorely needed. This study highlights the potential contribution of clothing to these interventions. The study also considers the potential contribution of religiosity to body appreciation, an issue that receives little attention compared to the extensive literature on the benefits of religion for mental health (Akrawi et al., 2015).

### 5.1 Limitations

Although the current study extended previous research, there are several limitations that need to be acknowledged. Firstly, the study lacked a control group or a follow-up, which means caution is warranted when drawing conclusions from the results. Secondly, the study relied on self-reports by MA students who were aware of the course’s feminist body-positive ideology. This awareness could have influenced their responses, introducing the possibility of bias. The missing data on BMI suggests that self-perception of weight should be measured in other ways, maybe by asking “how would you describe your body size.” Future research could also benefit from incorporating qualitative data to delve deeper into the nature of changes in body appreciation. Additionally, in the context of religion and body appreciation, it would be valuable to directly evaluate media exposure.Nevertheless, this research demonstrated the role of clothing in body appreciation and shed light on a developing field of research in the psychology of clothing.

## Data availability statement

The raw data supporting the conclusions of this article will be made available by the authors, without undue reservation.

## Ethics statement

The studies involving humans were approved by Academic College of Society and the Arts and its institutional research committee. The studies were conducted in accordance with the local legislation and institutional requirements. The participants provided their written informed consent to participate in this study.

## Author contributions

TS: Writing – original draft, Writing – review & editing.

## References

[ref1] Adam H. Galinsky A. D. (2012). Enclothed cognition. J. Exp. Soc. Psychol. 48, 918–925. doi: 10.1016/j.jesp.2012.02.008

[ref2] Akrawi D. Bartrop R. Potter U. Touyz S. (2015). Religiosity, spirituality in relation to disordered eating and body image concerns: a systematic review. J. Eat. Disord. 3:29. doi: 10.1186/s40337-015-0064-0, PMID: 26279837 PMC4536728

[ref4] Apeagyei P. R. (2008). Significance of body image among UK female fashion consumers: the cult of size zero, the skinny trend. Inter. J. Fash. Des. Tech. Edu. 1, 3–11. doi: 10.1080/17543260701867697

[ref5] Avalos L. Tylka T. L. Wood-Barcalow N. (2005). The body appreciation scale: development and psychometric evaluation. Body Image 2, 285–297. doi: 10.1016/j.bodyim.2005.06.002, PMID: 18089195

[ref6] Belk R. W. (1997). “Been there, done that, bought the souvenirs: of journeys and boundary crossing” in Consumer research: postcards from the edge. eds. Brown S. Turley D. (London: Routledge), 22–45.

[ref7] Benson A. L. (2000). I shop therefore I am. USA: Rowman & Littlefield publishers, Inc.

[ref01] Braizaz M. (2018). Femininity and fashion: How women experience gender role through their dressing practices. Cadernos Art. Antropol. 8, 59–76.

[ref8] Callis C. (1982). Appearance programs with female chronic psychiatric hospital patients: a comparison of six-week and nine-week treatment interventions. J. Rehab. 48, 34–39, PMID: 6891403

[ref9] Cash T. F. (2004). Body image: past, present, and future. Body Image 1, 1–5. doi: 10.1016/S1740-1445(03)00011-1 18089136

[ref10] Cash T. (2008). The body image workbook: an eight-step program for learning to like your looks. Oakland, CA: New Harbinger Publications.

[ref11] Cash T. L. Santos M. T. Williams E. F. (2005). Coping with body-image threats and challenges: validation of the body image coping strategies inventory. J. Psychosom. Res. 58, 190–199. doi: 10.1016/j.jpsychores.2004.07.008, PMID: 15820848

[ref12] Christel D. A. (2014). It’s your fault you’re fat: judgements of responsibility and social conduct in the fashion industry. Cloth. Cul. 1, 303–320. doi: 10.1386/cc.1.3.303_1

[ref13] Colls R. (2004). ‘Looking alright, feeling alright’: emotions, sizing and the geographies of women's experiences of clothing consumption. Soc. Cul. Geo. 5, 583–596. doi: 10.1080/1464936042000317712

[ref02] Costa P. T. McCrae R. R. (1992). Normal personality assessment in clinical practice: the NEO Personality inventory. Psychol. Assess. 4, 5–13. doi: 10.1037/1040-3590.4.1.5

[ref14] Engeln R. Zola A. (2021). These boots weren’t made for walking: gendered discrepancies in wearing painful, restricting, or distracting clothing. Sex Roles 85, 463–480. doi: 10.1007/s11199-021-01230-9, PMID: 34426714 PMC8373606

[ref03] Faul F. Erdfelder E. Lang A. G. Buchner A. (2007). G* Power 3: A flexible statistical power analysis program for the social, behavioral, and biomedical sciences. Behav. Res. Methods 39, 175–191. doi: 10.3758/BF03193146 17695343

[ref04] Fredrickson B. L. Roberts T. A. (1997). Objectification theory. Psychol Women Q. 21, 173–206. doi: 10.1111/j.1471-6402.1997.tb00108.x

[ref16] Frith H. Gleeson K. (2008). Dressing the body: the role of clothing in sustaining body pride and managing body distress. Qual. Res. Psychol. 5, 249–264. doi: 10.1080/14780880701752950

[ref17] Geller S. Handelzalts J. Gelfat R. Arbel S. Sidi Y. Levy S. (2020). Exploring body image, strength of faith, and media exposure among three denominations of Jewish women. Curr. Psychol. 39, 1774–1784. doi: 10.1007/s12144-018-9876-9

[ref18] Gilbert P. (2015). The evolution and social dynamics of compassion. Soc. Personal. Psychol. Compass 9, 239–254. doi: 10.1111/spc3.12176

[ref19] Guest E. Costa B. Williamson H. Meyrick J. Halliwell E. Harcourt D. (2019). The effectiveness of interventions aiming to promote positive body image in adults: a systematic review. Body Image 30, 10–25. doi: 10.1016/j.bodyim.2019.04.002 31077956

[ref20] Guy A. Banim M. (2000). Personal collections: women’s clothing use and identity. J. Gend. Stud. 9, 313–327. doi: 10.1080/713678000

[ref21] Halliwell E. (2013). The impact of thin idealized media images on body satisfaction: does body appreciation protect women from negative effects? Body Image 10, 509–514. doi: 10.1016/j.bodyim.2013.07.004 23972728

[ref22] Handelzalts J. E. Geller S. Levy S. Vered T. Fisher S. (2017). Body image among three denominations of Jewish women in Israel. Int. J. Cult. Ment. Health 10, 206–216. doi: 10.1080/17542863.2017.1290126

[ref26] Kaiser S. B. (1985). The social psychology of clothing. USA: Macmillan Publishing Company.

[ref28] Kang T. Y. Kim M. Y. (2021). Development of fashion therapy programs for improving body image and self-esteem. Res. J. Cost. Cult. 29, 167–184. doi: 10.29049/rjcc.2021.29.2.167

[ref29] Kim K. H. (2006). Religion, body satisfaction and dieting. Appetite 46, 285–296. doi: 10.1016/j.appet.2006.01.006, PMID: 16546296

[ref30] Koksal M. H. (2014). Psychological and behavioral drivers of male fashion leadership. Asia Pac. J. Market. Log. 26, 430–449. doi: 10.1108/APJML-06-2013-0067

[ref31] Kwon Y.-H. Parham E. S. (1994). Effects of state of fatness perception on weight conscious women’s clothing practices. Cloth. Text. Res. J. 12, 16–21. doi: 10.1177/0887302X9401200403

[ref32] Kwon Y. H. Shim S. (1999). A structural model for weight satisfaction, self-consciousness and women’s use of clothing in mood enhancement. Cloth. Text. Res. J. 17, 203–212. doi: 10.1177/0887302X9901700404

[ref33] Lee S. E. Lee Y. Yoo J. J. (2020). Understanding the fashion therapy (FT) experience through the cognitive behavioral perspective on body image. Inte. J. Cost. Fashion 20, 1–10. doi: 10.7233/ijcf.2020.20.2.001

[ref34] Lemma A. (2014). Minding the body: the body in psychoanalysis and beyond. London: Routledge.

[ref36] Masuch C. S. Hefferon K. (2014). Understanding the links between positive psychology and fashion: a grounded theory analysis. Inter. J. Fash. Stud. 1, 227–246. doi: 10.1386/infs.1.2.227_1

[ref37] McNeill L. Venter B. (2019). Identity, self-concept and young women’s engagement with collaborative, sustainable fashion consumption models. Inter. J. Cons. Stud. 43, 368–378. doi: 10.1111/ijcs.12516

[ref38] Moody W. Kinderman P. Sinha P. (2010). An exploratory study: relationships between trying on clothing, mood, emotion, personality and clothing preference. J. Fash. Market. Manag. 14, 161–179. doi: 10.1108/13612021011025483

[ref39] Murnen S. K. Seabrook R. (2012). “Feminist perspectives on body image and physical appearance,” in Encyclopedia of body image and human appearance. ed. Cash T. (Cambridge: Academic Press), 438–443.

[ref40] Mussap A. J. (2009). Acculturation, body image, and eating behaviors in Muslim-Australian women. Health Place 15, 532–539. doi: 10.1016/j.healthplace.2008.08.008 18952486

[ref41] Neff K. (2003). Development and validation of a scale to measure self-compassion. Self Iden. 2, 223–250. doi: 10.1080/15298860309027

[ref42] Peters L. D. (2014). You are what you wear: how plus-size fashion figures in fat identity formation. Fash. Theory 18, 45–71. doi: 10.2752/175174114X13788163471668

[ref43] Peterson R. D. Tantleff-Dunn S. Bedwell J. S. (2006). The effects of exposure to feminist ideology on women's body image. Body Image 3, 237–246. doi: 10.1016/j.bodyim.2006.05.004, PMID: 18089226

[ref44] Piran N. (2016). Embodied possibilities and disruptions: the emergence of the experience of embodiment construct from qualitative studies with girls and women. Body Image 18, 43–60. doi: 10.1016/j.bodyim.2016.04.007, PMID: 27236476

[ref45] Rahman O. Fung B. C. M. Kharb D. (2021). Factors influencing consumer choice: a study of apparel and sustainable cues from Canadian and Indian consumers' perspectives. Inter. J. Fash. Des. Tech. Edu. 14, 151–161. doi: 10.1080/17543266.2021.1898681

[ref46] Roach M. E. Eicher J. (1965). Dress, adornment and the social order. Americ. Soc. Rev. 31:896,

[ref47] Rocamora A. (2017). Mediatization and digital media in the field of fashion. Fash. The. 21, 505–522. doi: 10.1080/1362704X.2016.1173349

[ref48] Rodgers R. F. Laveway K. Campos P. de Carvalho P. H. B. (2023). Body image as a global mental health concern. Glob. Ment. Health 10:e9. doi: 10.1017/gmh.2023.2 PMC9970735 36861019

[ref49] Rudd N. A. Lennon S. J. (2000). Body image and appearance-management behaviors in college women. Cloth. Text. Res. J. 18, 152–162. doi: 10.1177/0887302X0001800304

[ref50] Seigelshifer V. Hartman T. (2011). From Tichels to hair bands: modern orthodox women and the practice of head covering. Women's Stud. Int. Forum 34, 349–359. doi: 10.1016/j.wsif.2011.05.006

[ref51] Shim S. Kotsiopulos A. Knoll D. S. (1990). Short, average-height, tall, and big men: body-cathexis, clothing and retail satisfactions, and clothing behavior. Percept. Mot. Skills 70, 83–96. doi: 10.2466/pms.1990.70.1.83

[ref52] Snyder R. Hasbrouck L. (1996). Feminist identity, gender traits, and symptoms of disturbed eating among college women. Psychol. Women Q. 20, 593–598. doi: 10.1111/j.1471-6402.1996.tb00324.x

[ref53] Sontag M. S. Lee J. (2004). Proximity of clothing to self scale. Cloth. Text. Res. J. 22, 161–177. doi: 10.1177/0887302X0402200402

[ref54] Srebnik D. Saltzberg E. A. (1994). Feminist cognitive-behavioral therapy for negative body image. Women Ther. 15, 117–133. doi: 10.1300/J015v15n02_10

[ref55] Stolovy T. (2021). Styling the self: clothing practices, personality traits, and body image among Israeli women. Front. Psychol. 12:3962. doi: 10.3389/fpsyg.2021.719318 PMC8455911 34566800

[ref56] Taragin-Zeller L. (2014). Modesty for Heaven’s sake: authority and creativity among female ultra-orthodox teenagers in Israel. Nashim Jew. Women Stud. Gen. Iss. 26, 75–96. doi: 10.2979/nashim.26.75

[ref57] Tiggemann M. (2004). Body image across the adult life span: stability and change. Body Image 1, 29–41. doi: 10.1016/S1740-1445(03)00002-0, PMID: 18089139

[ref58] Tiggemann M. Andrew R. (2012). Clothing choices, weight, and trait self-objectification. Body Image 9, 409–412. doi: 10.1016/j.bodyim.2012.02.003, PMID: 22465473

[ref59] Tiggemann M. Lacey C. (2009). Shopping for clothes: body satisfaction, appearance investment, and functions of clothing among female shoppers. Body Image 6, 285–291. doi: 10.1016/j.bodyim.2009.07.002, PMID: 19660999

[ref61] Tylka T. L. Piran N. (2019). Handbook of positive body image and embodiment: constructs, protective factors, and interventions. NY: Oxford University Press, 22–32.

[ref62] Tylka T. L. Wood-Barcalow N. L. (2015). What is and what is not positive body image? Conceptual foundations and construct definition. Body Image 14, 118–129. doi: 10.1016/j.bodyim.2015.04.001, PMID: 25921657

[ref63] Valaei N. Nikhashemi S. R. (2017). Generation Y consumers’ buying behaviour in fashion. Apparel industry: a moderation analysis. J. Fash. Market. Manag. 21, 523–543. doi: 10.1108/JFMM-01-2017-0002

[ref64] Wong S. E. Flanagan S. G. Kuehnel T. G. Liberinan R. P. Hunnicutt R. Adams-Badgert J. (1988). Training chronic mental patients to independently practice personal grooming skills. Psych. Serv. 39, 874–879. doi: 10.1176/ps.39.8.874, PMID: 3209205

[ref65] Yager Z. (2019). “Promoting positive body image and embodiment in schools: past, present, and future” in Handbook of positive body image and embodiment: constructs, protective factors, and interventions. eds. Tylka T. L. Piran N. (NY: Oxford University Press), 346–359.

